# Supplementary material for: E-selectin-mediated rapid NLRP3 inflammasome activation regulates S100A8/S100A9 release from neutrophils via transient gasdermin D pore formation
Source: Nat Immunol. 2023 Oct 30;24(12):2021–31. doi: 10.1038/s41590-023-01656-1 (PMC10681899; doi:10.1038/s41590-023-01656-1)

nature immunology

Article https://doi.org/10.1038/s41590-023-01656-1

E-selectin-mediated rapid NLRP3 inflammasome activation regulates S100A8/S100A9 release from neutrophils via transient gasdermin D pore formation

In the format provided by the authors and unedited

Table of contents

- **Supplementary Video 1**
- **Supplementary Video 2**
- **Supplementary Data (uncropped Western Blots)**
- **Reporting Summary**

uncropped western blots:

Figure 2a

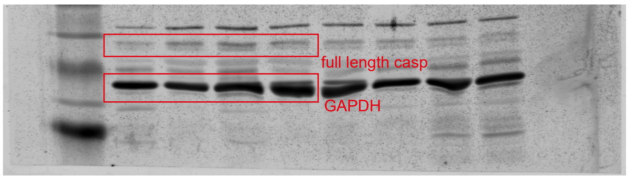

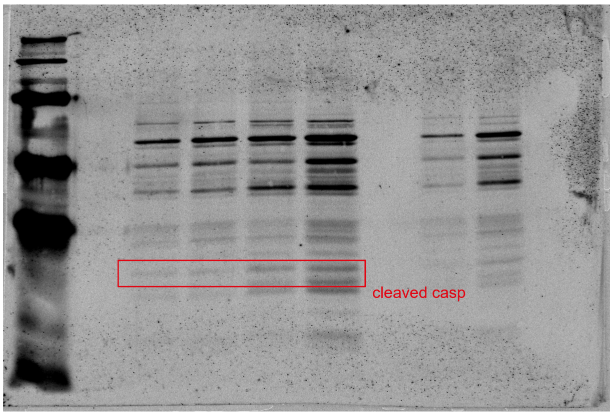

Figure 2b

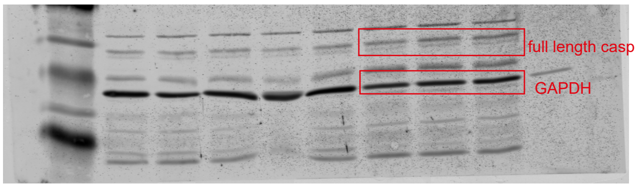

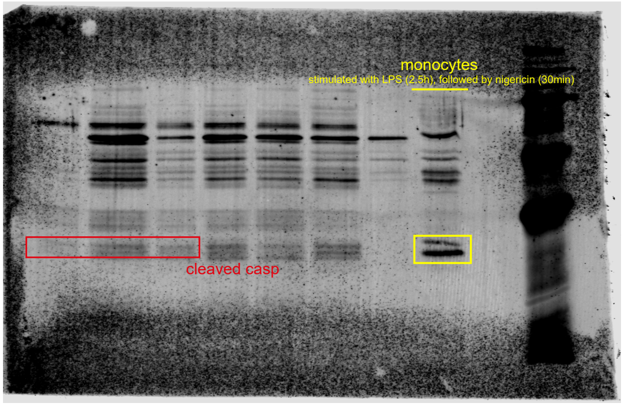

Figure 2f/Supplementary Figure 3a

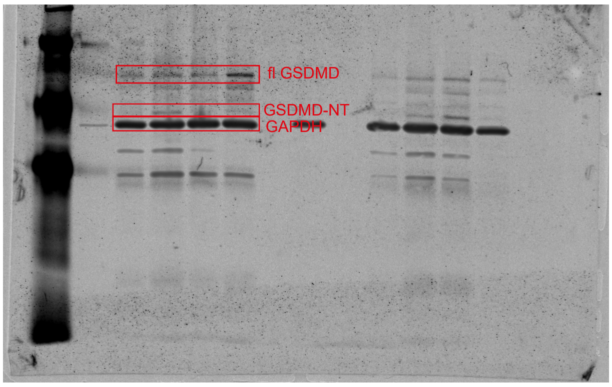

uncropped western blots:

Figure 3a

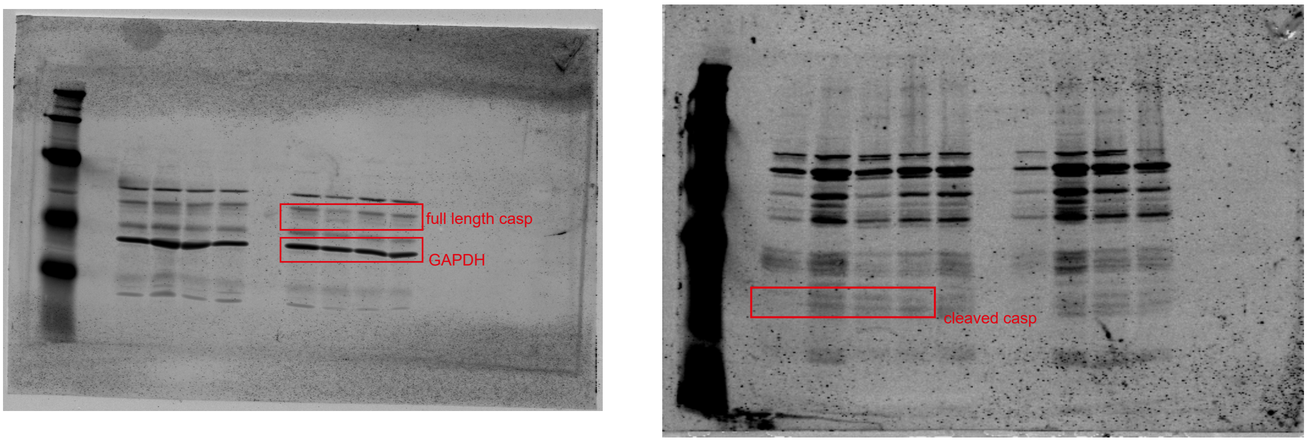

Figure 3b

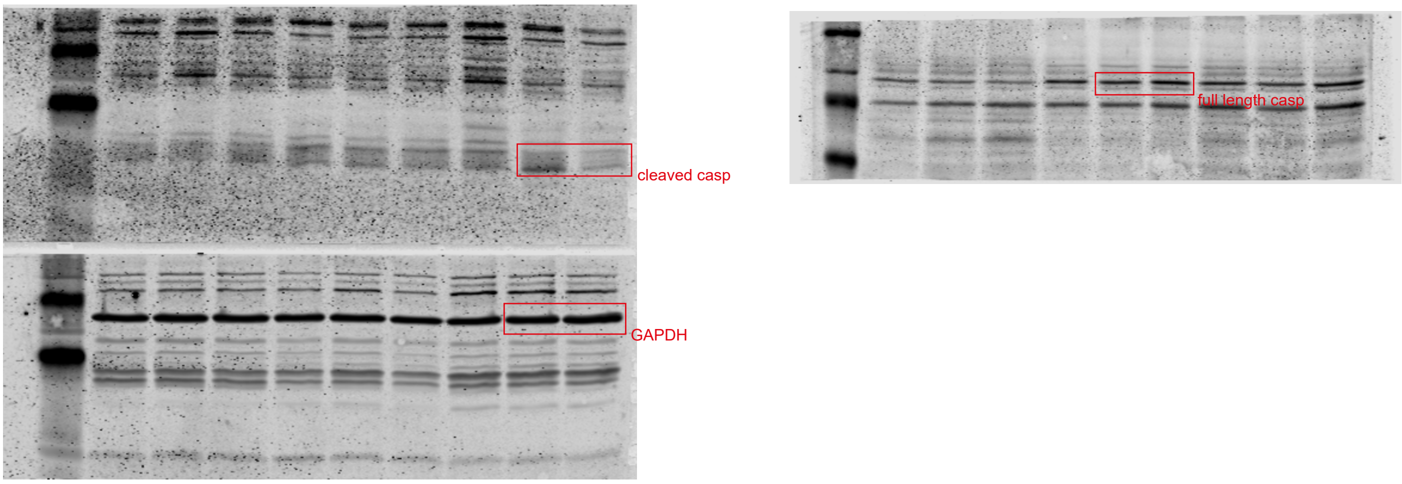

Figure 4a

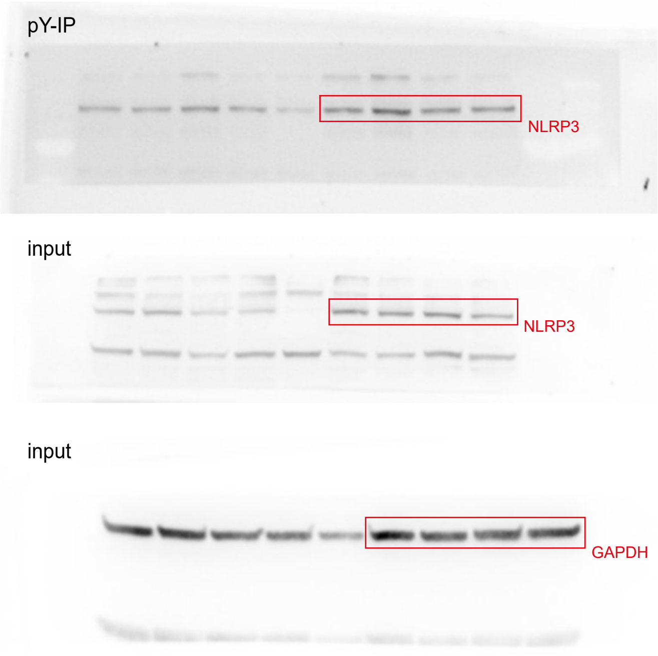

Figure 4c

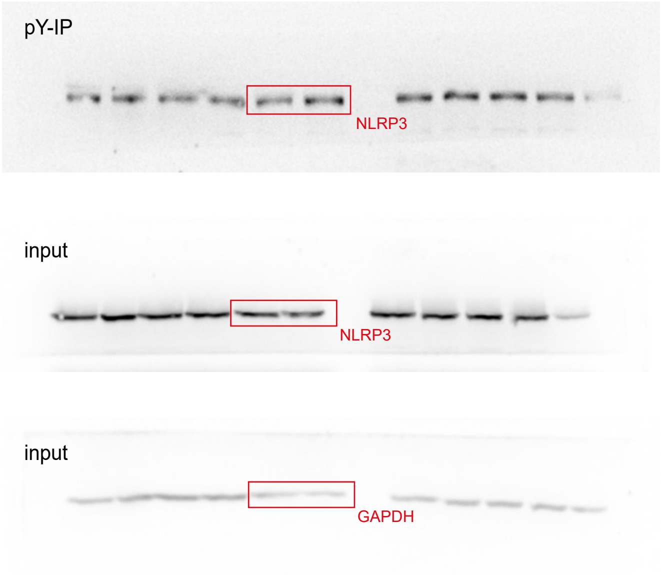

uncropped western blots:

Figure 4c and4f

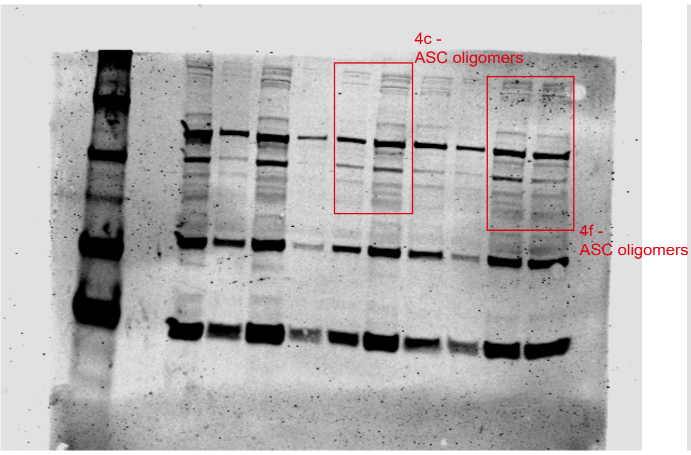

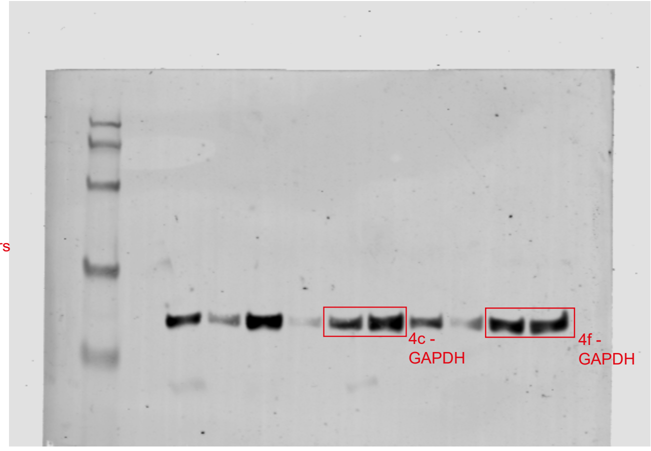

Supplementary Figure 1h

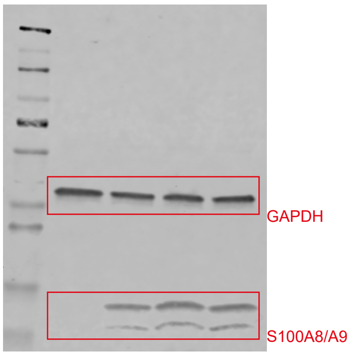

Supplementary Figure 2a

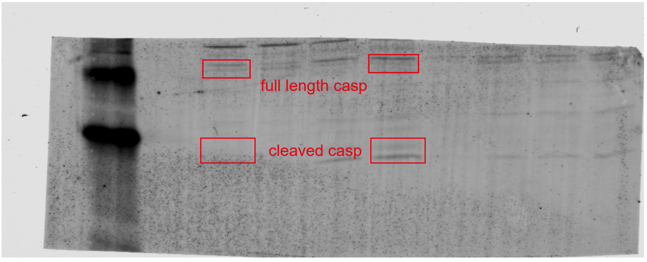

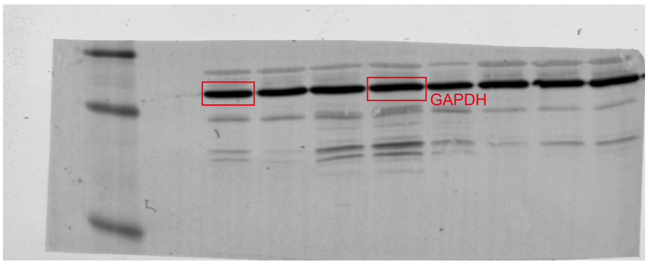

Supplement: Supplementary file 1 — Supplementary data. [file 41590_2023_1656_MOESM1_ESM.pdf]
