# Supplementary material for: E-selectin-mediated rapid NLRP3 inflammasome activation regulates S100A8/S100A9 release from neutrophils via transient gasdermin D pore formation
Source: Nat Immunol. 2023 Oct 30;24(12):2021–31. doi: 10.1038/s41590-023-01656-1 (PMC10681899; doi:10.1038/s41590-023-01656-1)
Supplement: Supplementary file 2 — Reporting Summary [file 41590_2023_1656_MOESM2_ESM.pdf]

nature portfolio

Corresponding author(s): Markus Sperandio

Last updated by author(s): Sep 8, 2023

Reporting Summary

Nature Portfolio wishes to improve the reproducibility of the work that we publish. This form provides structure for consistency and transparency in reporting. For further information on Nature Portfolio policies, see our Editorial Policies and the Editorial Policy Checklist.

Statistics

For all statistical analyses, confirm that the following items are present in the figure legend, table legend, main text, or Methods section.

n/a	Confirmed	
☐	☒	The exact sample size (n) for each experimental group/condition, given as a discrete number and unit of measurement
☐	☒	A statement on whether measurements were taken from distinct samples or whether the same sample was measured repeatedly
☐	☒	The statistical test(s) used AND whether they are one- or two-sided<br>*Only common tests should be described solely by name; describe more complex techniques in the Methods section.*
☒	☐	A description of all covariates tested
☐	☒	A description of any assumptions or corrections, such as tests of normality and adjustment for multiple comparisons
☐	☒	A full description of the statistical parameters including central tendency (e.g. means) or other basic estimates (e.g. regression coefficient) AND variation (e.g. standard deviation) or associated estimates of uncertainty (e.g. confidence intervals)
☐	☒	For null hypothesis testing, the test statistic (e.g. F, t, r) with confidence intervals, effect sizes, degrees of freedom and P value noted<br>*Give P values as exact values whenever suitable.*
☒	☐	For Bayesian analysis, information on the choice of priors and Markov chain Monte Carlo settings
☒	☐	For hierarchical and complex designs, identification of the appropriate level for tests and full reporting of outcomes
☒	☐	Estimates of effect sizes (e.g. Cohen's d, Pearson's r), indicating how they were calculated

Our web collection on statistics for biologists contains articles on many of the points above.

Software and code

Policy information about availability of computer code

Data collection

Western blots were scanned using the Odyssey® CLx Imaging System (LI-COR); MetaMorph software was used to generate rolling velocity movies; Flow cytometry was performed using a Beckman Coulter Gallios flow cytometer or CytoFLEX S, both Beckman Coulter; LDH levels and Il1beta in the supernatants were measured using aTecan SPARK 10M microplate reader. Patch clamp recordings were conducted using a HEKA EPC10 USB patch-clamp amplifier (HEKA Elektronik, Germany) in combination with Patchmaster software (v2x91). Postcapillary venules of the mouse cremaster muscle were recorded using a Olympus WX51 intravital microscope and VirtualDub software (version 1.10.4). Flow chamber experiments were conducted on a Zeiss Axiovert 200 microscope and MetaMorph software (version 6.2r6). Analysis of neutrophil extravasation was carried out on a Leica DM2500 microscope. Fluorescence images/time laps movies were aquired on a Leica SP8X WLL confocal microscope, equipped with a STED module using LAS X software (Leica, version 3.5 and newer). Mass spectometry was carried out using a quadrupole orbitrap tandem mass spectrometer (Orbitrap Exploris 480).

Data analysis

Blots were analyzed with Image Studio software (LI-COR; Lite version 5.2); Patch clamp recordings were analyzed with Origin Pro 2019G (version 9.6.0.172). Leukocyte rolling velocities, rolling flux fraction, number of adherent cells/mm2, vessel diameter and vessel length was determined on the basis of the generated movies using ImageJ software (version 1.52v) including M-TrackJ Plugin (version 1.5.1). Fluorescence images/time laps movies were analyzed using ImageJ software (version 1.52v). Flow cytometry data were analyzed using Kaluza (version 1.2) or Flowjo software (version 10.7 and newer). Mass spectometry data quantified with the R plugin DIA-NN (version 1.8.1) and statistically analyzed with the Perseus computational plattform (version 1.6.15). GraphPad Prism software (GraphPad Software Inc., version 7.05 and newer) and Adobe Illustrator (version 27.8) were used for statistical analyses and Figure design.

For manuscripts utilizing custom algorithms or software that are central to the research but not yet described in published literature, software must be made available to editors and reviewers. We strongly encourage code deposition in a community repository (e.g. GitHub). See the Nature Portfolio guidelines for submitting code & software for further information.

Data

Policy information about availability of data

All manuscripts must include a data availability statement. This statement should provide the following information, where applicable:

- Accession codes, unique identifiers, or web links for publicly available datasets
- A description of any restrictions on data availability
- For clinical datasets or third party data, please ensure that the statement adheres to our policy

All data that support the findings of this study are available from the corresponding author upon reasonable request. DIA MS/MS spectra were searched against the Uniprot SWISSPROT human proteome (version from 2023/02/15). The mass spectrometry proteomics data have been deposited to the ProteomeXchange Consortium via the PRIDE partner repository with the dataset identifier PXD041652. Source data are provided with this paper.

Research involving human participants, their data, or biological material

Policy information about studies with human participants or human data. See also policy information about sex, gender (identity/presentation), and sexual orientation and race, ethnicity and racism.

Reporting on sex and gender	Human neutrophils were isolated from healthy male and female donors. Due to low sample numbers we did not differentiate between male and female.
Reporting on race, ethnicity, or other socially relevant groupings	Human neutrophils were isolated from healthy male and female donors. Due to low sample numbers we did not differentiate between race, ethnicity or social relevant groupings.
Population characteristics	Blood samples were taken from healthy volunteers 20-40 years of age.
Recruitment	Recruitment of healthy volunteers was randomly performed at the Biomedical Center at LMU Munich, Germany, through announcements at the institute's dash board. Informed consent was obtained from all participants.
Ethics oversight	Blood sampling from healthy volunteers was approved by the ethical committee of Ludwig-Maximilians-Universität München, Munich, Germany (Az. 611-15) in agreement with the Declaration of Helsinki.

Note that full information on the approval of the study protocol must also be provided in the manuscript.

Field-specific reporting

Please select the one below that is the best fit for your research. If you are not sure, read the appropriate sections before making your selection.

☒ Life sciences ☐ Behavioural & social sciences ☐ Ecological, evolutionary & environmental sciences

For a reference copy of the document with all sections, see nature.com/documents/nr-reporting-summary-flat.pdf

Life sciences study design

All studies must disclose on these points even when the disclosure is negative.

Sample size	We have not performed any sample size power calculations. In our studies, we based the sample size on optimization experiments or previously conducted experiments (in vivo and in vitro experiments) and used a minimum of 3-10 individual samples per group. Published sample sizes from our group in the literature include (Kurz AR, Pruenster M, Rohwedder I, Ramadass M, Schäfer K, Harrison U, Gouveia G, Nussbaum C, Immler R, Wiessner JR, Margraf A, Lim DS, Walzog B, Dietzel S, Moser M, Klein C, Vestweber D, Haas R, Catz SD, Sperandio M. MST1-dependent vesicle trafficking regulates neutrophil transmigration through the vascular basement membrane. J Clin Invest. 2016 Nov 1;126(11):4125-4139. doi: 10.1172/JCI87043) and (Pruenster M, Kurz AR, Chung KJ, Cao-Ehlker X, Bieber S, Nussbaum CF, Bierschenk S, Eggersmann TK, Rohwedder I, Heinig K, Immler R, Moser M, Koedel U, Gran S, McEver RP, Vestweber D, Verschoor A, Leanderson T, Chavakis T, Roth J, Vogl T, Sperandio M. Extracellular MRP8/14 is a regulator of β2 integrin-dependent neutrophil slow rolling and adhesion. Nat Commun. 2015 Apr 20;6:6915. doi: 10.1038/ncomms7915) and (Rohwedder I, Kurz ARM, Pruenster M, Immler R, Pick R, Eggersmann T, Klapproth S, Johnson JL, Alsina SM, Lowell CA, Mócsai A, Catz SD, Sperandio M. Src family kinase-mediated vesicle trafficking is critical for neutrophil basement membrane penetration. Haematologica. 2020 Jul;105(7):1845-1856. doi: 10.3324/haematol.2019.225722)
Data exclusions	No data was excluded.
Replication	All experiments were performed at least three times and were successfully replicated. The number of experiments is indicated in the respective figure legends.
Randomization	Mice of similar age and sex were used for all experiments except for intravital microscopy studies in the cremaster muscle, where male mice were used only. Allocation of animals to groups was performed randomly or on the basis of their genotype. Human blood samples, all obtained from healthy volunteers (please also refer to Recruitment) were allocated randomly to groups with no relevant covariates.
Blinding	Most of the conducted experiments were performed in a non-blinded fashion but kept as unbiased as possible. Individual in vivo and in vitro

Blinding

experimental series contained appropriate internal controls and normalization methods (as described in Methods) and were conducted by the same researcher to guarantee reproducibility. In vitro S100A8/A9 release assays were analyzed in a blinded fashion using numerical keys for sample and group allocation.

Reporting for specific materials, systems and methods

We require information from authors about some types of materials, experimental systems and methods used in many studies. Here, indicate whether each material, system or method listed is relevant to your study. If you are not sure if a list item applies to your research, read the appropriate section before selecting a response.

Materials & experimental systems

n/a	Involved in the study	
☐	☒	Antibodies
☒	☐	Eukaryotic cell lines
☒	☐	Palaeontology and archaeology
☐	☒	Animals and other organisms
☒	☐	Clinical data
☒	☐	Dual use research of concern
☒	☐	Plants

Methods

n/a	Involved in the study	
☒	☐	ChIP-seq
☐	☒	Flow cytometry
☒	☐	MRI-based neuroimaging

Antibodies

Antibodies used

1) polyclonal rabbit anti-mouse MRP8 (provided by Thomas Vogl, Muenster, Germany, 1µg/ml)
2) polyclonal rabbit anti-mouse MRP14 (provided by Thomas Vogl, Muenster, Germany, 5µg/ml)
3) mouse anti GAPDH (Calbiochem, #CB1001, clone 6C5, 0.15µg/ml)
4) goat anti-rabbit IRDye 800CW (LI-COR Bioscience, #926-3211, 0.1µg/ml)
5) goat anti-mouse IRDye 680RD (LI-COR Bioscience, #926-68070, 0.1µg/ml)
6) polyclonal rabbit anti human caspase-1 (Cell Signaling, #2225S, 1:1000)
7) polyclonal rabbit anti human GSDMD (Cell Signaling, #96458S, 1:2000)
8) rabbit anti-human cleaved n-terminal (NT-)GSDMD antibody (Abcam, #ab215203, clone EPR20829-408, 2µg/ml)
9) donkey anti-rabbit Alexa488 (Thermo Fisher, #A21206, Lot: 802706, 5µg/ml)
10) polyclonal rabbit anti human CHMP4B (abcam, #AB135154, Lot: GR3424084-3, 5µg/ml)
11) goat anti rabbit abberior STAR 635P (Abberior, #2-0012-007-2; Lot: 19092016Hp, 2µg/ml)
12) polyclonal rabbit anti mouse ASC (Adipogen, #AG-25B-0006-C100, Lot. A42092103, clone AL177, 5µg/ml (microscopy), 1µg/ml (WB))
13) Phospho-Tyrosine MultiMab™ rabbit mAB mix (Cell Signaling, #14017, 1:25 (20µl))
14) mouse anti NLRP3 (Adipogen, #AG-20B-0014-C100, clone Cryo-2, 1:1000)
15) polyclonal goat anti-mouse HRP (Jackson Immuno Research, #155-035-003, 1:15000)
16) APC conjugated rat anti mouse CD11a (eBioscience, #170111, Lot:1911677, clone M17/4, 5µg/ml)
17) BV510 conjugated rat anti mouse CD11b (Biolegend #:101245, Lot: B261558, clone M1/70, 5µg/ml)
18) FITC conjugated rat anti mouse CD18 (Pharmingen, #553292, Lot:22531, clone C71/16, 5µg/ml)
19) APC conjugated rat anti mouse CXCR2 (R&D Systems, #FAB2164A, Lot: LMC0816111, clone 242216, 5µg/ml)
20) Pacific Blue conjugated rat anti mouse Ly6G (Biolegend, #127612, clone 1A8, 5µg/ml)
21) APC conjugated rat IgG2a, κ Isotype Ctrl (Biolegend, #400512, clone RT2758, 5µg/ml)
22) BV510 conjugated rat IgG2b, κ Isotype Ctrl (Biolegend, #400645, Lot: B167358, clone RTK4530, 5µg/ml)
23) FITC conjugated rat IgG2a, κ Isotype Ctrl (eBioscience, #11-4321-85, Lot: 7054789, clone eBR2a, 5µg/ml)
24) APC conjugated rat IgG2a, κ Isotype Ctrl (Biolegend, #400512, Lot: B238056, clone RTK2758, 5µg/ml)
25) FITC conjugated anti human CD63 (Biolegend, #353006, clone H5C6, 5µg/ml)
26) Pacific blue conjugated anti human CD66b (Biolegend, #305112, clone G10F5, 5µg/ml)
27) APC/Fire™ 750 conjugated mouse anti human CD11b (Biolegend, #301352, clone ICRF44, 5µg/ml)
28) FITC conjugated mouse IgG1, κ Isotype Ctrl (Biolegend, #400108, clone MOPC-21, 5µg/ml)
29) Pacific Blue mouse conjugated IgM, κ Isotype Ctrl (Biolegend, #401619, clone MM-30, 5µg/ml)
30) APC/Fire™ 750 conjugated mouse IgG1, κ Isotype Ctrl (Biolegend, #400196, clone MOPC-21, 5µg/ml)
31) APC conjugated anti human CD15 (Biolegend, #323007, clone W6D3, 5µg/ml)

Validation

Numbering refers to the list of antibodies above:
1) Used as reported in: (Pruenster M, Kurz AR, Chung KJ, Cao-Ehlker X, Bieber S, Nussbaum CF, Bierschenk S, Eggersmann TK, Rohwedder I, Heinig K, Immler R, Moser M, Koedel U, Gran S, McEver RP, Vestweber D, Verschoor A, Leanderson T, Chavakis T, Roth J, Vogl T, Sperandio M. Extracellular MRP8/14 is a regulator of β2 integrin-dependent neutrophil slow rolling and adhesion. Nat Commun. 2015 Apr 20;6:6915. doi: 10.1038/ncomms7915)
2) See 1)
3) https://www.merckmillipore.com/DE/de/product/Anti-GAPDH-Mouse-mAb-6C5,EMD_BIO-CB1001?ReferrerURL=https%3A%2F%2Fwww.google.com%2F#anchor_PDS
4) https://www.licor.com/documents/rfm2hw40wf33p06f3ndjrcorwi5usbft
5) https://www.licor.com/documents/7bohf1sfzugccz22fh0um00cvz8ocizf
6) https://www.cellsignal.com/datasheet.jsp?productId=2225&images=1
7) https://www.cellsignal.com/datasheet.jsp?productId=96458&images=1
8) https://www.abcam.com/cleaved-n-terminal-gsdmd-antibody-epr20829-408-ab215203.html
9) https://www.thermofisher.com/antibody/product/Donkey-anti-Rabbit-IgG-H-L-Highly-Cross-Adsorbed-Secondary-Antibody-

Polyclonal/A-21206
10) https://www.abcam.com/chmp4b-antibody-ab135154.html
11) https://www.sigmaaldrich.com/DE/en/product/sigma/53399
12) https://adipogen.com/ag-25b-0006-anti-asc-pab-al177.html
13) https://www.cellsignal.de/products/antibody-conjugates/phospho-tyrosine-p-tyr-1000-multimab-rabbit-mab-mix-magnetic-bead-conjugate/14017
14) https://adipogen.com/ag-20b-0014-anti-nlrp3-nalp3-mab-cryo-2.html
15) https://www.jacksonimmuno.com/catalog/products/115-035-003
16) https://www.thermofisher.com/antibody/product/CD11a-LFA-1alpha-Antibody-clone-M17-4-Monoclonal/17-0111-80
17) https://www.biolegend.com/de-de/products/brilliant-violet-510-anti-mouse-human-cd11b-antibody-7993
18) https://www.bdbiosciences.com/en-us/products/reagents/flow-cytometry-reagents/research-reagents/single-color-antibodies-ruo/fitc-rat-anti-mouse-cd18.553292
19) https://www.rndsystems.com/products/mouse-cxcr2-il-8rb-apc-conjugated-antibody-242216_fab2164a
20) https://www.biolegend.com/de-de/products/pacific-blue-anti-mouse-ly-6g-antibody-6082
21) https://www.biolegend.com/de-de/products/apc-rat-igg2a-kappa-isotype-ctrl-1838
22) https://www.biolegend.com/de-de/products/brilliant-violet-510-rat-igg2b-kappa-isotype-ctrl-8018
23) https://www.thermofisher.com/antibody/product/Rat-IgG2a-kappa-clone-eBR2a-Isotype-Control/11-4321-85
24) https://www.biolegend.com/en-gb/products/apc-rat-igg2a-kappa-isotype-ctrl-1838
25) https://www.biolegend.com/de-de/products/fitc-anti-human-cd63-antibody-7434
26) https://www.biolegend.com/de-de/products/pacific-blue-anti-human-cd66b-antibody-9583
27) https://www.biolegend.com/de-de/products/apc-fire-750-anti-human-cd11b-antibody-13561
28) https://www.biolegend.com/de-de/products/fitc-mouse-igg1-kappa-isotype-ctrl-1406
29) https://www.biolegend.com/de-de/products/pacific-blue-mouse-igm-kappa-isotype-ctrl-3167
30) https://www.biolegend.com/de-de/products/apc-fire-750-mouse-igg1-kappa-isotype-ctrl-13011
31) https://www.biolegend.com/de-de/products/apc-anti-human-cd15-ssea-1-antibody-3702

Animals and other research organisms

Policy information about studies involving animals; ARRIVE guidelines recommended for reporting animal research, and Sex and Gender in Research

Laboratory animals	C57BL/6NCrl (WT) mice were purchased from Charles River Laboratories (Sulzfeld, Germany). Gsdmd-/- and Casp1-/-Casp11-/- mice were obtained from Petr Broz, Epalinges, Switzerland and Veit Hornung, Munich, Germany, respectively. Mrp14-/- were provided by Johannes Roth, Muenster, Germany. Sele-/- were provided by Dietmar Vestweber, Muenster, Germany. All mice, including Kcna3-/- mice were housed at the Biomedical Center, LMU, Planegg-Martinsried, Germany. 8-25 weeks old male and female mice were used for all experiments.<br>Room temperature and relative humidity ranged from 20 to 22°C and from 45–55%, respectively. The light cycle was adjusted to 12h light:12 h dark period. Room air was exchanged 11 times per hour and filtered with HEPA-systems. All mice were housed in individually ventilated cages (TypII long, Tecniplast, Germany) under specified-pathogen-free conditions. Hygiene monitoring was performed every three months based on recommendations of the FELASA-14 working group. All animals had free access to water and food (irradiated, 10mm pellet; 1314P, Altromin, Netherlands). The cages were equipped with nesting material (5 × 5cm, Nestlet, Datesand, UK), a red corner house (Tecniplast, Germany) and a rodent play tunnel (7.5 × 3.0cm, Datesand, UK). Soiled bedding (LASbedding, 3–6mm, PG3, Las vendi, Germany) was removed every 7 days.
Wild animals	No wild animals were used
Reporting on sex	Male and female mice were used for all experiments except for intravital microscopy studies in the cremaster muscle, where male mice were used only. Allocation of animals to groups was performed on the basis of their genotype.
Field-collected samples	Our study did not involve samples collected from the field.
Ethics oversight	Animal experiments were approved by the Government of Oberbayern (AZ.: ROB-55.2-2532.Vet_02-18-22 and ROB-55.2-2532.Vet_02-17-102) and carried out in accordance with the guidelines from Directive 2010/63/EU.

Note that full information on the approval of the study protocol must also be provided in the manuscript.

Flow Cytometry

Plots

Confirm that:

☒ The axis labels state the marker and fluorochrome used (e.g. CD4-FITC).

☒ The axis scales are clearly visible. Include numbers along axes only for bottom left plot of group (a 'group' is an analysis of identical markers).

☒ All plots are contour plots with outliers or pseudocolor plots.

☒ A numerical value for number of cells or percentage (with statistics) is provided.

Methodology

Sample preparation	Human neutrophils were isolated from healthy blood donors using Polymorphprep (Axis Shield) or EasySepTM human

Sample preparation	neutrophil direct isolation kit (STEMCELL TECHNOLOGIES) according to manufacturer's protocols; Bone marrow mouse neutrophils were isolated using EasySepTM mouse neutrophil enrichment kit (STEMCELL TECHNOLOGIES) according to manufacturer's protocol.
Instrument	Flow cytometry was performed using a Beckman Coulter Gallios flow cytometer or CytoFLEX S, both Beckman Coulter.
Software	Data were analyzed using Kaluza (version 1.2) or Flowjo software (version 10.7 and newer)
Cell population abundance	Neutrophils were definded as CD15 positive (human) and Ly6G positive (murine) population. Purity after isolation was approx. 75-80% (murine samples) and approx. 90-95% (human samples).
Gating strategy	Neutrophils were definded as CD15 positive (human samples) and Ly6G positive (murine) population.

☒ Tick this box to confirm that a figure exemplifying the gating strategy is provided in the Supplementary Information.
